# Non-technical skill performance during remote, international, augmented-reality neonatal resuscitation protocol simulations: a feasibility study

**DOI:** 10.1186/s12909-026-08611-2

**Published:** 2026-02-05

**Authors:** Marcos Rojas, Romy Yun, Asheen Rama, Jaime Plane, Claudia Arancibia, Pamela Paredes, Antonello Penna, Yiling Zhao, Thomas J. Caruso

**Affiliations:** 1 Stanford School of Medicine, Stanford University, California, United States; 2 Graduate School of Education, Stanford University, California, United States; 3 Facultad de Medicina, Universidad de Chile, Santiago, Chile; 4 Departamento de Educación en Ciencias de la Salud, Facultad de Medicina, Universidad de Chile, Santiago, Chile; 5 Departamento de Anestesiología y Medicina Perioperatoria, Hospital Clínico de la Universidad de Chile, Santiago, Chile; 6 Centro de Investigación Clínica Avanzada (CICA), Facultad de Medicina, Hospital Clínico de la Universidad de Chile, Santiago, Chile

**Keywords:** Augmented reality, Non-technical skills, Neonatal resuscitation, Simulation, Medical education

## Abstract

**Background:**

Neonatal resuscitation requires mastery of technical and non-technical skills (NTS). The Neonatal Resuscitation Program (NRP) emphasizes ventilation, along with effective leadership, teamwork, and situational awareness. Simulation-based education is central to NTS training but is resource-intensive with limited access in low- and middle-income countries. Augmented reality (AR) medical simulations offer a scalable, remote solution with fewer logistical barriers.

**Methods:**

We conducted a prospective, single-arm feasibility study evaluating a remote AR medical simulation for assessing NTS during neonatal resuscitation. Instructors based in Northern California (Stanford School of Medicine) remotely facilitated pediatric and anesthesiology residents in Santiago, Chile (University of Chile) as team leaders in an NRP scenario delivered using an AR medical simulator. The primary outcome was NTS performance measured with the Anesthetists’ Non-Technical Skills (ANTS) tool. Secondary outcomes included the Behaviorally Anchored Rating Scale (BARS), system usability via the System Usability Scale (SUS), and ergonomics via the ISO 9241 − 400 scale.

**Results:**

Thirty-four residents completed all sessions. Participants demonstrated adequate NTS performance, with ANTS scores 2.5–2.9 (scale 1–4) and BARS scores 5.2–5.4 (scale 1–9), similar to those observed in traditional, in-person simulations. SUS responses indicated high usability, and ergonomic results suggested minimal physical or cognitive burden.

**Conclusions:**

This feasibility study shows that participants demonstrated measurable NTS performance during remote, international AR neonatal resuscitation simulations, as assessed using ANTS and BARS.

**Supplementary Information:**

The online version contains supplementary material available at 10.1186/s12909-026-08611-2.

## Background

Neonatal resuscitation is a rare and critical event, requiring mastery of both technical and non-technical skills (NTS). To support the unique physiology of newborns, the Neonatal Resuscitation Program (NRP) provides a crisis-management algorithm, distinct from pediatric and adult resuscitation [1]. Adherence to NRP during medical crises improves neonatal survival with fewer complications [2, 3]. Low- and middle-income countries (LMICs) experience disproportionately higher rates of poor neonatal outcomes, highlighting the need to expand access to neonatal resuscitation training [4–6].

Simulation education is central to NRP training, allowing teams to practice critical scenarios in structured and safe environments [7–10]. However, disadvantages to traditional simulation include significant resource allocation such as cost, equipment, space, and expert facilitators [11–13]. Although technical components of NRP can be practiced on manikins, NTS such as leadership, situational awareness, and decision-making are best observed in high-fidelity, team-based simulations [14]. In LMIC settings, limited access to simulation resources and expert facilitation constrains opportunities to develop and maintain these skills [15].

Extended reality (XR) is a term that includes virtual reality (VR) and augmented reality (AR). VR places the learner in a fully virtual environment, whereas AR overlays virtual content upon the natural, real world, preserving interaction with real people and physical equipment [16]. This “pass-through” feature is particularly relevant to non-technical skills, which depend on real-time verbal and non-verbal communication, situational observation, information gathering, and coordination among team members during a crisis.

Medical simulations remotely conducted with AR can increase access to high-fidelity, expert-facilitated simulation by reducing reliance on onsite facilitators and specialized infrastructure [11–13, 16]. Through experiential learning, AR simulations partially immerse participants in crises that support leadership and communication behaviors [17] as well as crisis task management and decision-making in resuscitation scenarios [18], while maintaining in-room team interaction [19, 20]. AR simulations have shown favorable usability and low ergonomic burden in pediatric and adult resuscitation training [21, 22], while published applications of remote AR simulation for neonatal resuscitation remain limited, particularly in LMIC contexts [23].

Prior to potentially using remote AR simulations in LMICs, we designed this feasibility investigation to study the progression of learners’ NTS with an international instructor in a resourced setting to identify opportunities and strengths of this teaching modality. This sequencing follows scale-up frameworks that recommend iterative piloting and context adaptation before broader implementation [24]. The goal of this feasibility study was to evaluate whether participants successfully progressed through a remote, international AR simulation focused on neonatal resuscitation. The primary outcome investigated progression through the simulated neonatal crisis by examining learners’ NTS. Secondary outcomes explored further assessment of NTS, system usability, and ergonomics.

We selected a synchronous, real-time remote AR format because many NTS behaviors are expressed and observable only during live team interaction. In this format, learners remain physically co-located with their team and equipment while a remote instructor shares the same clinical visual context (e.g., holographic patient and monitor) and facilitates scenario progression in real time. This combination of AR and real-time communication is intended to mitigate geographic barriers to expert-led neonatal resuscitation simulation and inform future implementation in resource-limited settings [11–13, 16, 19, 20, 23].

## Methods

### Study design

This prospective, single-arm study evaluated the use of remote, international AR simulation for assessing NTS performance during neonatal resuscitation. The study was conducted at two collaborating sites: Lucile Packard Children’s Hospital Stanford (LPCHS, Stanford, California, USA) and the University of Chile (Santiago, Chile), where the simulation instructors and pediatric and anesthesiology residents were located, respectively.

Ethical approval was obtained from both institutions. At Stanford, approval was granted under protocol IRB-70,882. At the University of Chile, approval was granted under Proyecto Nº 198–2023, Acta Nº 182. The study was registered at ClinicalTrials.gov (NCT05906173) on June 7, 2023.

This manuscript adheres to the TREND (Transparent Reporting of Evaluations with Nonrandomized Designs) statement for nonrandomized interventional studies [25].

### Participants and setting

Pediatric and anesthesiology residents from any year of training were enrolled at the University of Chile. All participants were recruited from a single institution, where training follows a standardized curriculum. In Chile, trainees complete a seven-year undergraduate medical program followed by a three-year residency program; enrolled residents were in years 1–3 of residency. We did not collect baseline NTS scores prior to the simulation. In the Chilean context, pediatric and anesthesiology residents have a curricular requirement to develop competencies in neonatal resuscitation, making them the primary trainees for whom simulation-based training is the most relevant. Since the simulation, orientation, and debriefing were conducted in English, inclusion required an English proficiency level of at least B2 according to the Common European Framework of Reference for Languages [26]. Residents were excluded if they had a history of seizures, severe motion sickness, current nausea, or if they used corrective glasses incompatible with the AR headset. Recruitment occurred through electronic communications distributed by residency program administrators, and enrollment proceeded on a convenience basis as residents met eligibility requirements. All sessions were conducted in the Clinical Skills Center at the University of Chile, with simulation instructors remotely located at LPCHS and connected through the AR software.

### Intervention

All participants completed a single AR neonatal resuscitation simulation using the Stanford Chariot AR medical (CHARM) simulator (Stanford Chariot Program, Stanford, CA) which integrates real-time communication into a portable AR medical simulator featuring a holographic patient, monitor, and other medical equipment. The simulation scenario was scripted according to the NRP algorithm [27] (Supplemental materials A and B) and incorporated the full sequence of steps, including: warming, drying, stimulation through positive pressure ventilation, corrective maneuvers using the MR SOPA mnemonic (Mask adjustment, Reposition airway, Suction mouth and nose, Open mouth, Pressure increase, Alternative airway), advanced airway management, chest compressions, and epinephrine administration.

The scenario and remote communications between the instructors and participants were delivered through the CHARM simulator software on the Magic Leap One (ML1, Plantation, FL) AR headset, which projected holographic clinical elements, including the neonatal patient and vital signs monitor, as well as resuscitation equipment (e.g., bag-mask device, defibrillator, and IV supplies), into the physical room (Supplemental material C). Each resident participated individually in the role of team leader, supported by two actors who played a receiving nurse and a respiratory therapist. The two actors were trained and standardized on the scenario script prior to study initiation. Their role was to provide consistent team interactions that elicited observable NTS (e.g., communication and coordination) while executing technical actions only when directed by the participant, without coaching clinical decisions. Simulation instructors based at LPCHS remotely facilitated case progression and controlled the holographic imagery, while actors and research assistants at the University of Chile provided onsite support.

Each session consisted of an orientation, a preparatory video, the AR simulation, a debriefing, and post-intervention questionnaires. The orientation, conducted by a research assistant in Chile, introduced participants to the study and its procedures, obtained informed consent, and collected demographic information. During this phase, residents were also familiarized with the AR headsets and the holographic elements they would interact with during the simulation. Following the orientation, participants watched a five-minute video that reviewed the NRP algorithm, introduced the clinical case and scenario context, and outlined the responsibilities of the team leader role with examples of specific tasks to be performed. The simulation itself, which lasted approximately 10–12 min, was then conducted. A research assistant trained in NTS rating observed participants continuously throughout the scenario. After the simulation, participants engaged in a structured five-minute debriefing led by the same instructor who facilitated the simulation, focusing on guided reflection regarding their clinical performance and decision-making processes [28]. Finally, participants completed post-intervention questionnaires. NTS ratings were based on performance during the simulation only (prior to debriefing). Post-intervention questionnaires assessed system usability and ergonomics and were not intended to measure NTS performance. The total time commitment per participant, including all components, was approximately one hour. No financial or material incentives were provided for participation.

### Objectives

The objective was to evaluate whether residents successfully progressed through a remote, international AR simulation designed to assess NTS during a neonatal resuscitation scenario. The primary hypothesis was that participants would demonstrate typical NTS performance during the simulation compared to previously reported in-person NTS scoring of anesthesiology residency in their second through fourth year of training [29]. ANTS is widely used to assess NTS. Prior resident cohorts reported mean scores of 2.2 with a standard deviation (SD) of 0.7 [29]. Similarly, we classified these cohort means as typical and interpreted higher means as an educationally meaningful improvement. The secondary hypothesis was that the AR system would show acceptable usability and ergonomic burden, supporting its future implementation.

### Outcomes and measures

The primary outcome was NTS performance, assessed with the ANTS tool [30] (Supplemental material D). ANTS is a validated instrument designed to evaluate observable NTS in clinical settings. It comprises of four domains: task management, team working, situation awareness, and decision-making, each subdivided into specific skill elements anchored to behavioral descriptors. For each descriptor within a domain, raters assign a score on a four-point scale: 1 (poor), 2 (marginal), 3 (acceptable), and 4 (good), based on observed behaviors during the simulation.

Secondary outcomes included further evaluation of NTS, system usability, and ergonomic burden. NTS were evaluated using the Behaviorally Anchored Rating Scale (BARS) [31] (Supplemental material E), a validated tool that links specific behavioral examples to numerical ratings. BARS assesses four core domains relevant to crisis resource management: vigilance and situational awareness, decision-making, communication, and teamwork. Each domain is rated at one of three performance levels (poor, average, or excellent), with numerical scores assigned within defined ranges: 1–3 for poor, 4–6 for average, and 7–9 for excellent.

Usability of the AR system was measured using the System Usability Scale (SUS) [32] (Supplemental material F), a widely used 10-item questionnaire rated on a 5-point Likert scale from 1 (Completely Disagree) to 5 (Completely Agree), providing a global measure of perceived system usability. Ergonomic burden was evaluated with the ISO 9241 − 400 ergonomic scale [33] (Supplemental material G), which consists of six items rated on a 5-point Likert scale from 1 (Totally Disagree) to 5 (Totally Agree), capturing participants’ perceptions of physical comfort and interaction quality with the AR system.

ANTS and BARS were scored by the simulation instructor and a research assistant present during the scenario, both trained in NTS rating. Raters completed scoring independently and were blinded to each other’s ratings. Because this was a feasibility study, discrepancies were not adjudicated; for analysis, each participant’s ANTS and BARS scores were represented by the mean of the two raters’ ratings. Inter-rater reliability was not formally assessed. SUS and ergonomic outcomes were self-reported by participants after the simulation. All instruments were administered electronically through REDCap (Research Electronic Data Capture, Vanderbilt University, Nashville, TN, USA) [34].

### Sample size

Sample size calculations were based on previously reported ANTS scores among residents (mean 2.2, SD 0.7) [29], with a 30% improvement to 2.9 defined as educationally meaningful. Assuming α = 0.05 and 80% power, the required sample was 32 participants. No interim analyses were planned.

### Assignment and blinding

This was a non-randomized, single-arm feasibility study; therefore, no randomization was performed. To standardize scenario delivery, we used a pre-specified script that defined the sequence of events and instructor prompts, including when changes in the holographic patient and vital signs were triggered. Participants were aware of their role as team leader, and simulation instructors and research assistants could not be blinded. ANTS and BARS assessments were completed independently by the simulation instructor and a research assistant trained in NTS rating. The research assistant’s role was limited to observation and scoring and did not include facilitation or coaching.

### Statistical methods

Descriptive statistics were used to summarize participant demographics and outcome measures. For ANTS and BARS, performance for each participant was represented by the mean of the two scorers’ ratings. SUS and ISO results were summarized at the item level. All analyses were conducted in RStudio (version 2023.03.0 + 386; RStudio Team, 2024).

## Results

### Participant characteristics

A total of 36 residents were enrolled in the study between August and December 2024. Two did not meet the language requirement and were excluded, leaving 34 who completed the intervention and all surveys. Of these, 7 were men and 27 were women, representing both pediatrics and anesthesiology residency programs at the University of Chile (Table 1). Residency year and prior simulation and AR exposure to contextualize baseline experience relevant to simulation performance were recorded (Table 1).

**Table 1:** Demographics of study participants

Demographics*	Values (*n* = 34)
Age, mean (SD)	30.4 (3.0)
Female, n (%)	27 (79.4%)
Race**	
American Indian or Alaskan Native	1 (2.9%)
Asian	0 (0.0%)
Black or African American	1 (2.9%)
Native Hawaiian or Other Pacific	0 (0.0%)
White	23 (67.6%)
Prefer not to answer	5 (14.7%)
Other	5 (14.7%)
Ethnicity	
Hispanic or Latino	34 (100.0%)
Year in Residency Training	
First year	12 (35.3%)
Second year	15 (44.1%)
Third year	7 (20.6%)
Number of prior in-person simulations	
0	14 (41.2%)
1–2	11 (32.4%)
3–5	3 (8.8%)
6–10	4 (11.8%)
> 10	2 (5.9%)
Number of prior AR experiences	
0	33 (97.1%)
1–2	0 (0.0%)
3–5	0 (0.0%)
6–10	1 (2.9%)
> 10	0 (0.0%)

*For continuous variables, values are presented as mean (standard deviation); for categorical variables, values are presented as number (percentage). **Multiple answers allowed

### Primary outcome

Participants demonstrated typical performance across all four ANTS domains, with mean scores ranging from 2.5 to 2.9 on the 1–4 scale (Table 2). Task management was rated between 2.7 and 2.8 across elements, while teamwork scored highest overall, particularly in coordinating activities and exchanging information (mean 2.9, SD 0.9 for both). Situational awareness showed greater variability, with the lowest scores observed for anticipating (mean 2.5, SD 0.9). Decision-making element scores ranged from 2.6 to 2.8.

**Table 2:** ANTS ratings by domain and element

Domain and Element	Mean (SD)
Task Management	
Planning & preparing	2.8 (0.8)
Prioritizing	2.7 (0.8)
Providing & maintaining standards	2.7 (0.8)
Identifying & utilizing resources	2.8 (0.9)
Team Working	
Coordinating activities with team	2.9 (0.9)
Exchanging information	2.9 (0.9)
Using authority & assertiveness	2.7 (0.9)
Assessing capabilities	2.6 (0.8)
Situation Awareness	
Supporting others	2.8 (0.8)
Gathering information	2.9 (0.8)
Recognizing & understanding	2.7 (0.9)
Anticipating	2.5 (0.9)
Decision Making	
Identifying options	2.8 (0.8)
Balancing risks & selecting options	2.8 (0.8)
Re-evaluating	2.6 (0.8)

### Secondary outcomes

**Fig. 1:**
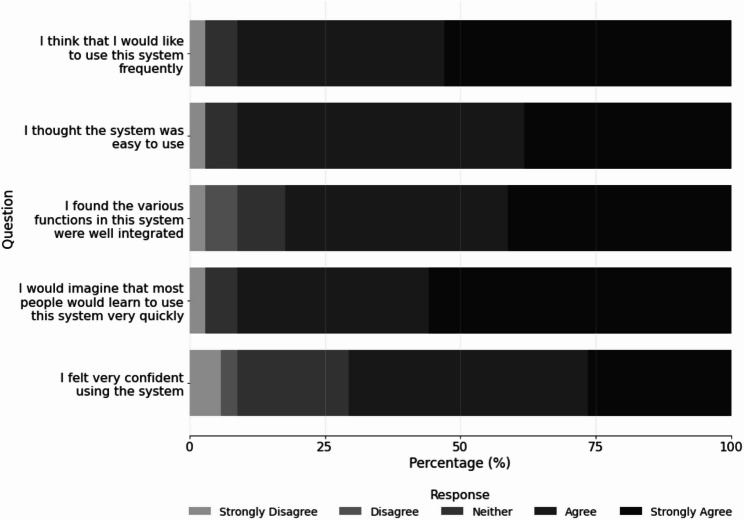
Responses to positive items of the System Usability Scale (SUS)

**Fig. 2:**
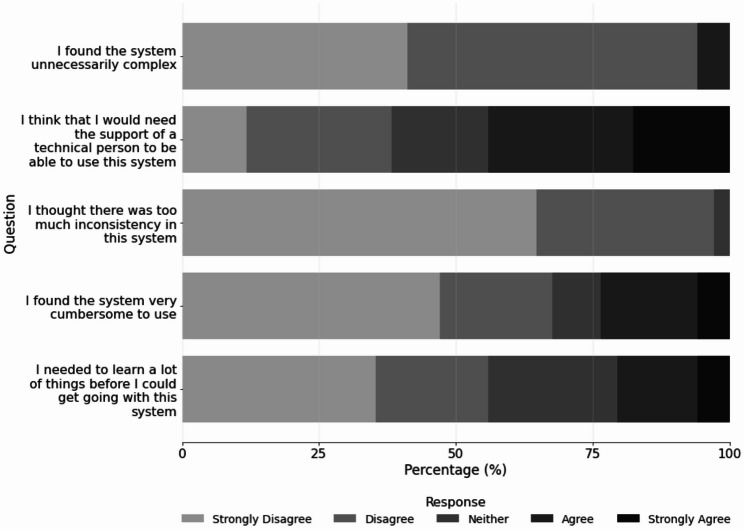
Responses to negative items of the System Usability Scale (SUS)

Participants showed consistent performance across BARS domains, with mean scores ranging from 5.2 to 5.4 (Table 3). Usability, assessed with SUS, demonstrated that most participants agreed or strongly agreed that most users would likely learn to use the system quickly (91.2%), that the AR system was easy to use (91.1%), suitable for repeated use (91.1%), well-integrated (82.4%), and something they felt confident operating (70.6%) (Fig. 1). Participants generally disagreed or strongly disagreed with negative statements about the system, with most indicating that it did not have inconsistencies (97.1%), was not unnecessarily complex (94.1%), and was not cumbersome to use (67.7%). A smaller majority disagreed or strongly disagreed that they would need to learn many things before getting started (55.9%). The only item that generated mixed responses concerned whether technical support would be needed to operate the system, with 44.1% of participants agreeing or strongly agreeing with that statement (Fig. 2).

**Table 3:** BARS ratings by domain

Domain	Mean (SD)
Vigilance	5.3 (2.0)
Decision Making	5.2 (2.1)
Communication	5.4 (2.0)
Teamwork	5.4 (2.0)

Regarding ergonomics, most participants disagreed or strongly disagreed with statements indicating that the device caused arm fatigue (94.1%), led to head fatigue (94.1%), caused eye fatigue (91.1%), was too heavy (82.4%), or required very high mental effort to operate (76.5%). In contrast, most participants (70.6%) agreed or strongly agreed that the device could be worn comfortably for extended use (Supplemental material H).

### Adverse events

Adverse events were defined as any participant-reported symptoms related to AR headset use during the session, including nausea, dizziness, motion sickness, headache, visual discomfort/eye strain, or other physical discomfort. No participants reported any such adverse effects during the intervention.

## Discussion

This study evaluated NTS performance during remote, international AR simulations of neonatal resuscitation in a high-resource country. Participants successfully progressed through the AR scenario, demonstrating typical performance across the ANTS domains of task management, teamwork, situation awareness, and decision making [29]. Observed ANTS scores (2.5–2.9 on a 1–4 scale) were similar to previously reported scores for residents assessed in in-person simulation settings [29, 35]. NTS performance was consistent when assessed with the complementary BARS instrument, reinforcing the validity of the primary results. In addition, usability scores indicated that participants perceived the AR platform as intuitive and well-integrated into the training environment, while ergonomic responses suggested minimal physical or cognitive burden from the headset. These outcomes support remote AR simulations for assessing NTS, while also highlighting the platform’s acceptability and usability in an international setting.

Remote AR simulations achieved ANTS performance levels comparable to those during in-person simulations [35, 36]. Although NTS can improve following simulation across fidelity levels, high-fidelity manikin-based simulations with expert facilitation provide richer physiologic cues, time pressure, and team-role interdependence that can elicit and assess NTS more comprehensively [35]. Low-fidelity simulations can still support improvement in ANTS domains in LMIC settings [37] but may offer fewer clinical cues and less immersive team dynamics than high-fidelity formats. A synchronous remote AR format may plausibly help bridge this gap by preserving key high-fidelity elements (shared visual clinical context via holographic patient/monitor, real-time scenario control, and expert facilitation) while reducing reliance on onsite expert faculty and specialized infrastructure. However, this study was not designed to test whether AR is superior to low-fidelity or traditional high-fidelity simulation; comparative effectiveness and superiority claims require future randomized or controlled studies. Consistent with scale-up frameworks, these findings support iterative piloting and adaptation as precursors to broader LMIC implementation [24].

NTS, including situation awareness, decision making, teamwork, and task management, are increasingly recognized as critical components of safe and effective patient care [38, 39]. In resuscitation contexts, ANTS-based assessments correlate with meaningful performance outcomes, with higher scores associated with faster and more effective crisis resolution [35, 38]. Despite this, NTS have been overlooked in traditional medical training, particularly in crisis management contexts such as neonatal resuscitation, where clinical focus is placed on ventilation and other technical skills [40]. However, recent curricular developments in internal medicine and emergency medicine residencies are incorporating structured training in communication, leadership, and behavioral skills, providing a more holistic approach [41–43]. Similarly, accreditation bodies such as the Accreditation Council for Graduate Medical Education (ACGME) mandate inclusion of non-clinical competencies, emphasizing that excellence in healthcare requires more than technical expertise [44]. Remote AR simulation offers a promising approach to integrate NTS training into diverse curricula, providing efficient setup, immersive practice opportunities for team leaders, and the ability to extend training to environments without local experts.

The secondary outcomes of usability and ergonomics further support AR simulation for educational use. The high usability ratings are consistent with prior evaluations of AR platforms, including earlier applications of the CHARM simulator in pediatric and adult resuscitation scenarios [17, 21, 22]. Technological promise alone is insufficient if users perceive the headsets as cumbersome or impractical. The positive usability and ergonomic outcomes suggest that AR simulation could be integrated into training programs without significant barriers, aligning with broader frameworks such as the Technology Acceptance Model (TAM), which emphasizes perceived ease of use and usefulness as key drivers of adoption in graduate medical education [45].

The international collaboration between Stanford University and the University of Chile demonstrated educational viability across continents and time zones, highlighting the ability of remote, AR simulation to overcome geographic barriers. However, this study had several limitations. First, the single-arm design without a control group limits causal inference, and the small sample size restricts generalizability. Second, baseline NTS performance was not measured prior to the AR simulation, and we did not collect detailed neonatal-specific clinical experience beyond residency year and prior simulation exposure. Unmeasured participant differences could have influenced observed performance. Third, the study was conducted at a single international site, which may not capture participant variability across other institutions or cultural contexts. Fourth, convenience sampling with participant self-selection may have introduced selection bias in an unpredictable direction. Fifth, the Hawthorne effect cannot be excluded since participants were aware of being observed, potentially impacting performance. Sixth, the simulation instructors and facilitators were members of the research team, which may have introduced expectancy or observer bias in facilitation and scoring despite standardized scripts and independent ratings. Seventh, because this was a feasibility study, discrepancies between raters’ scores were not adjudicated; analyses used the mean of the two raters’ ANTS and BARS scores. Finally, the sample size calculation was based on a previously published resident cohort from a different institution; baseline ANTS scores may have differed from our participants, although observed performance was similar in magnitude to previously reported in-person simulation cohorts.

Future studies should incorporate larger sample sizes with multicenter participation and assess the impact of AR simulation on NTS retention. Rigorous comparison with high-fidelity traditional simulation would establish relative effectiveness. AR simulation is positioned to become a scalable and accessible approach to strengthen NTS training in neonatal resuscitation globally, with particular benefit to regions where traditional simulation resources remain limited.

## Conclusion

This international feasibility study successfully engaged residents in a remote, expert-facilitated AR neonatal resuscitation scenario and demonstrated that NTS performance can be assessed using validated instruments (ANTS and BARS) in this format. Usability and ergonomics findings indicated that participants perceived the platform as acceptable with minimal reported burden, supporting the practicality of remote AR simulation delivery across sites. Future studies should use controlled designs to evaluate training effects and to compare remote AR with low- and high-fidelity simulation approaches, while accounting for implementation constraints such as hardware and internet connectivity.

## Supplementary Information

Supplementary Material 1.

## Data Availability

The de-identified datasets and analysis code that support the findings of this study are available from the corresponding author (MR) on reasonable request and under a data-use agreement to protect participant privacy and institutional confidentiality. All instruments and scenario materials are provided in the supplementary files (Supplemental materials A–H).

## Abbreviations

ACGME: Accreditation Council for Graduate Medical Education
ANTS: Anaesthetists’ Non-Technical Skills
AR: Augmented reality
BARS: Behaviorally Anchored Rating Scale
CICA: Centro de Investigación Clínica Avanzada
CHARM: Stanford Chariot AR Medical (simulator)
IRB: Institutional Review Board
ISO: International Organization for Standardization
ISO 9241 − 400: International standard on ergonomics of human–system interaction (physical input devices)
LMICs: Low- and middle-income countries
LPCHS: Lucile Packard Children’s Hospital Stanford
ML1: Magic Leap One (augmented-reality headset)
MR SOPA: Mask adjustment, Reposition airway, Suction mouth and nose, Open mouth, Pressure increase, Alternative airway
NRP: Neonatal Resuscitation Program
NTS: Non-technical skills
REDCap: Research Electronic Data Capture
SD: Standard deviation
SUS: System Usability Scale
TREND: Transparent Reporting of Evaluations with Nonrandomized Designs
USA: United States of America
