# Supplementary material for: Non-technical skill performance during remote, international, augmented-reality neonatal resuscitation protocol simulations: a feasibility study
Source: BMC Med Educ. 2026 Feb 5;26:387. doi: 10.1186/s12909-026-08611-2 (PMC12973738; doi:10.1186/s12909-026-08611-2)

**Supplemental Material**

**Supplemental Material A. NRP Simulation Steps and Script**

Roles assigned in advance:

1. Stanford University:
   1. Clinical professor: Provides simulation session
   2. Two research assistants**:** Apply ANTS and BARS to measure NTS
2. Universidad de Chile:
   1. Participant: Team Leader/NRP provider with advanced airway skills
   2. Confederate 1: Receiving RN/basic NRP provider
   3. Confederate 2: Respiratory Therapist
   4. Research assistant: Connects participants to Zoom and CHARM

Order of Events:

1. Introduction and informed consent
2. Effective communication and teamwork information
3. Debrief with examples
4. AR simulation: 5-6min
5. Debrief about simulation
6. Questionnaires

Additional materials:

1. Physical space to perform AR clinical simulation
2. Clinical vignette
3. NRP Algorithm 8^th^ edition
4. 1 Newborn CPR manikin
5. 1 Table

Clinical Scenario:

You are called by your OB anesthesiology colleague who is taking care of a G1P0 patient who urgently went into the OR for an emergency Cesarean Delivery for prolapsed umbilical cord/fetal bradycardia. Mother is stable with working epidural and upon delivery with appropriate uterine tone. However, the baby, upon delivery, is limp with poor tone and is not breathing. The Pediatrics team has been called; however they are unavailable for another 5 minutes. Your OB anesthesia colleague is concerned the baby may need advanced resuscitation and has asked you to help the receiving RN with the baby.

The receiving RN, who is NRP certified, has initiated the 1^st^ step of NRP and has warmed, stimulated, and dried baby. RN has suctioned mouth and nose as well. However, baby is still limp and apneic. The RN has initiated positive pressure ventilation with a T-piece resuscitator however there is no chest rise.  You are told that the baby is term, not breathing and not crying. (can consider asking participant instead of mentioning these three things)

AR Simulation Steps

1. Instructor headset: create a room
   1. In AR activate the following elements
      1. Neonatal patient – on a warmer
      2. Vital signs monitor but with no vitals present
2. Prepare participant headset, check if Wi-Fi is working correctly, open CHARM Sim and enter room. Once inside the room:
   1. turn off microphone
   2. Anchoring complete
3. AR headset is placed on participant

Script:

Instructor: Tells participant that they have been called to provide assistance and care for a 1 minute-old limp and apneic term baby. Participant is told that the simulation team will conduct all procedures, they should remain seated and delegate tasks to the receiving nurse and respiratory therapist and that they are the “team lead”

Instructor: “the RN is attempting PPV however no chest rise is seen. Baby is still apneic, what do you want to do?”

Begin PPV within 60 seconds of birth

Within 15 seconds of beginning PPV at FiO2 21%, team leader/learner should ask RN to assess the HR and state if HR is increasing. Learner should ask RN to place monitors (EKG and pulse oximeter on baby). RN will place EKG and pulse oximeter monitors on (pulse ox on R hand/pre-ductal). Vital sign monitor should indicate HR at this point, unreadable pulse ox

Instructor: will turn on SaO2 and HR on vital signs monitor. “Heart rate is 60 and not increasing” “What is your next step?”

Assess chest movement

No chest movement is observed on holographic baby. Learner to instruct RN corrective steps of MR SOPA. (Mask adjustment, Reposition head, Suction mouth and nose, Open mouth, Pressure increase, and Alternative airway)

Instructor: “now you see chest movement with PPV, what is your next step in management?”

Check HR after 30 seconds of PPV that moves the chest

Learner asks to check HR and HR is 60bpm and not increasing

Instructor: “HR still 60bpm, what do you want to do next?” May need to prompt learner to start thinking about advanced airway

Insert alternative airway (ETT)

Learner should state what size ETT/blade. If not then RT asks what size ETT and what blade to intubate patient. RT then intubates baby

Instructor: “what do you confirm next?” “how do you confirm correct placement of ETT and position?”

Learner instructs RN to auscultate both sides of chest. Learner should ask for color change on CO2 detector.

Instructor: “ There are no breath sounds hears and no chest rise is seen” “what do you do now”

Learner recognizes esophageal intubation and re-intubates. Confirm color change on CO2 detector (color might not change on detector 2/2 low HR), bilateral breath sounds and visible chest rise.

Instructor: “you have a successful intubation--what do you check now”

Check HR after 30 seconds of PPV with alternative airway

Learner checks HR by looking at monitor. HR still 60bpm and not increasing. Pulse ox still with no signal

Instructor: “What do you do now?”

Begin chest compressions

Learner calls for additional help. Asks RT to increase FiO2 to 100%. Asks RN to start chest compressions at the head of the bed with coordinated ventilation (3 compressions: 1 ventilation)

Cadence: “one and two and three and breathe…”

Instructor: “your team has started chest compressions” “what do you want to check?”

Check HR after 60 seconds

Learner has team pause chest compressions but continues PPV and checks HR. HR is checked every 60 seconds of compressions and ventilation.

Instructor: “HR is still 60 bpm” “what should you consider?”

Learner indicates need for UVC access. At this point, additional help has arrived and is working on placing UVC access—however it is difficult.

Instructor: “what alternative option do you have for medication administration?” “the baby is roughly 3kg”

Medication administration via ETT while UVC being established

Learner indicates epi administration through ETT (100mcg/kg). Learner announceh medication. RN administers ETT epi. RN announces “endotracheal epi dose given.”

Prepare emergency UVC (or PIV)
Insert UVC (or PIV)

Instructor: “UVC/PIV line is now established, what do you want to do next?” If learner does not realize to give epi then should prompt with “Do you wait 3-5 minutes after the last ETT epi dose?”

Administer medications through UVC/PIV

Learner knows not to count the ETT epi dose and instructs RN to give appropriate dose of Epi through UVC. Learner should instruct RN in ml (not mcg). RN flushes med with 3ml of flush. Learner uses closed-loop communication with confirmation of medication, dose and route. RN announces “IV epi given.”

Instructor: “what do you check now?”

Check HR after 60 seconds

Learner instructs a pause on compressions/ventilation to check HR on monitor. HR is 60bpm. Learner instructs to continue compressions/ventilation

Instructor: “What is your next step?”

Administer volume expander

Learner requests 10ml/kg of volume expander (30ml) of NS through UVC line using closed loop communication. Compressions/ventilation still continuing.

Instructor: “what do you want to do next?”

Check HR after 60 seconds

Learner asks to stop compressions/ventilation to check HR. HR is 80 bpm and continuing to increase.

Instructor: “Next step?”

Discontinue chest compressions—continue PPV

Learner asks to stop chest compressions but continue ventilation (40-60 breaths/min—which is a higher ventilation rate vs during compressions). Monitor at this point should read HR=80bpm and SaO2=80%

Instructor: “There are still no spontaneous respirations.”

Check vital signs

Learner continues PPV and adjusts O2 concentration per pulse oximetry table. Monitor should then transition to HR= 120bpm and SaO2= 90%

Instructor: “There are some spontaneous respirations and baby has fair muscle tone”

End scenario

Learner supports baby with PPV and supplemental O2 per Target O2 Saturation Table. Monitors HR, respiratory effort, O2 sat, activity and temperature. Prepares to move baby to post-resuscitation care setting. Communicates with perinatal team. Updates parents and informs them of next steps. Debriefs the resuscitation.

**Supplemental Material B: Neonatal Resuscitation Algorithm. Outlined in the 8th edition of the Textbook of Neonatal Resuscitation (American Academy of Pediatrics and American Heart Association, 2021).**


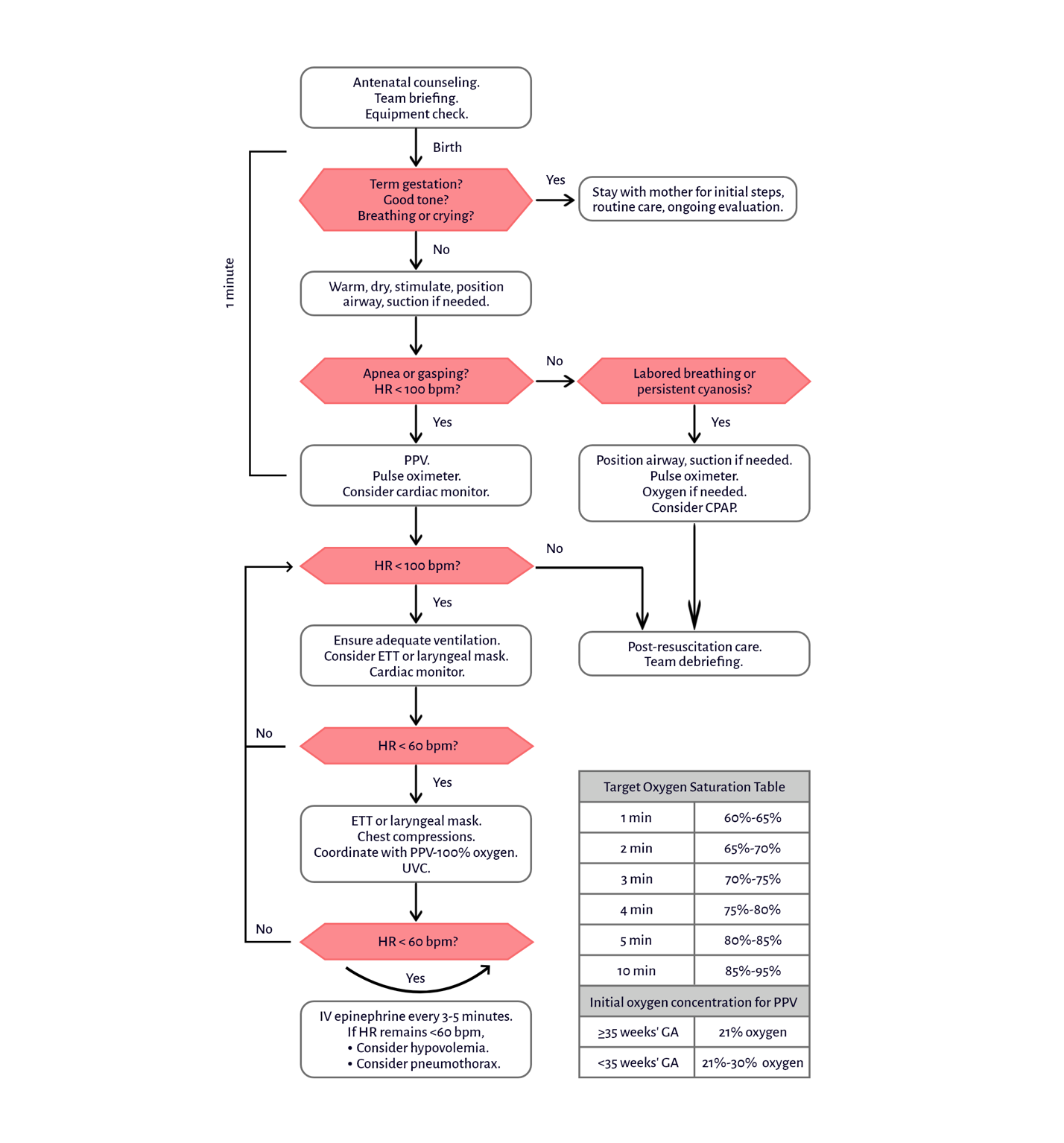


**Supplemental Material C: Example view of the AR simulation environment as seen through the Magic Leap One headset. The only physical elements in the room are the table/bed surface (with the manikin location) and the participants wearing headsets; the neonatal patient, vital signs monitor, and resuscitation equipment (e.g., bag-mask device, defibrillator, and IV supplies) are displayed as holographic projections.**


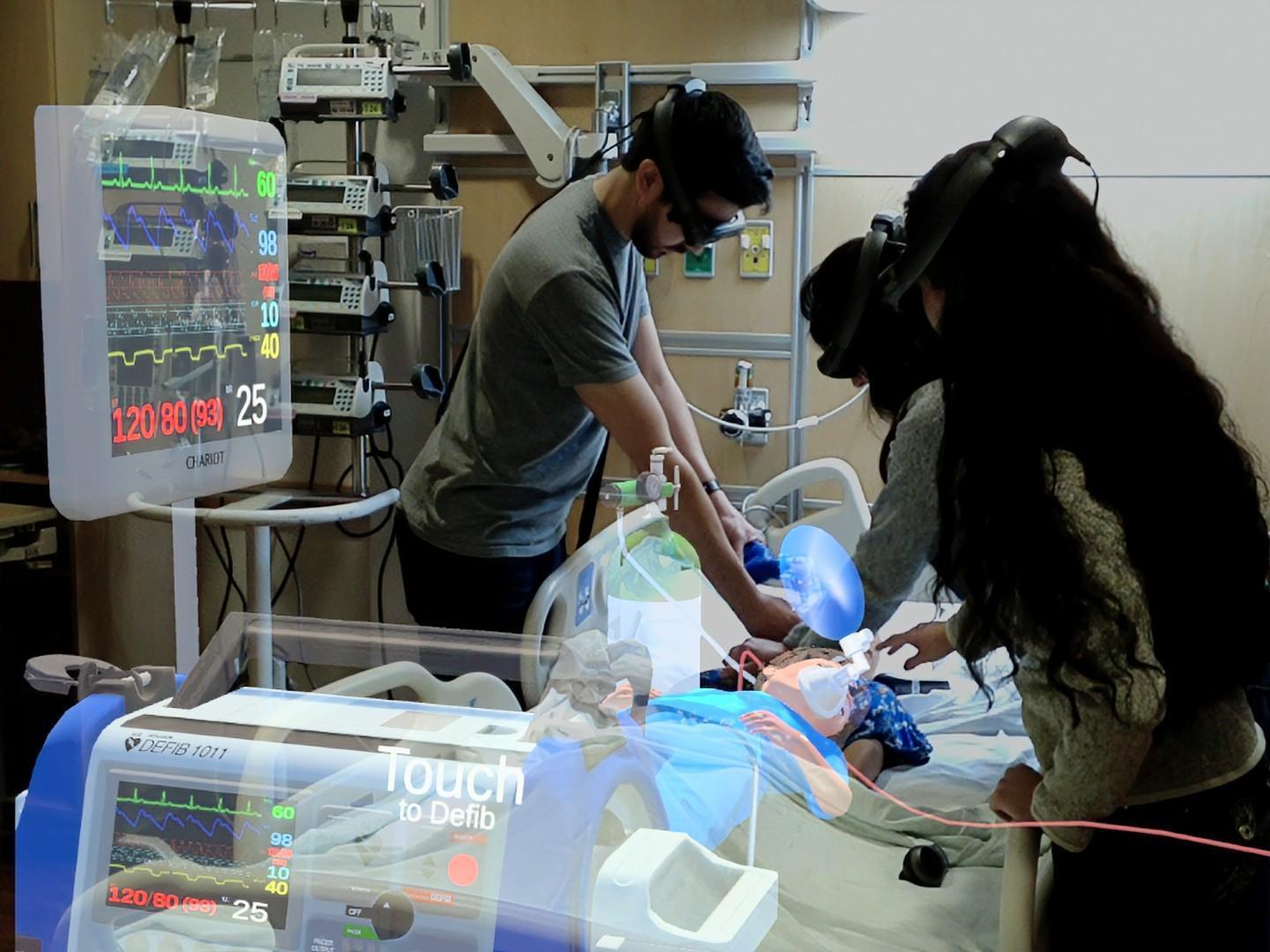


**Supplemental Material D: Anaesthetists’ Non-Technical Skills. Refer to this manuscript for additional details: DOI: 10.1007/s10111-004-0158-y**


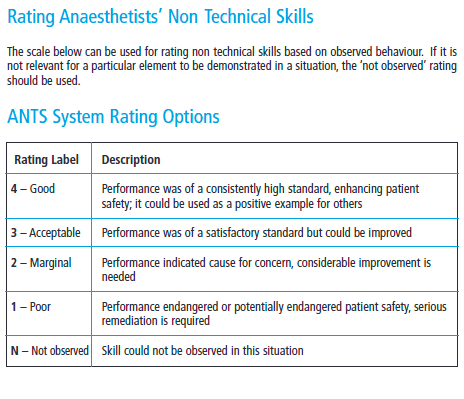


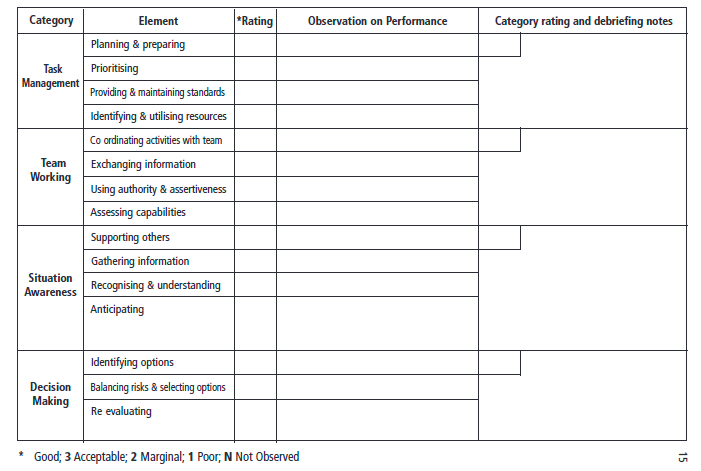


**Supplemental Material E: The Behavioral/Non-technical Rating System**


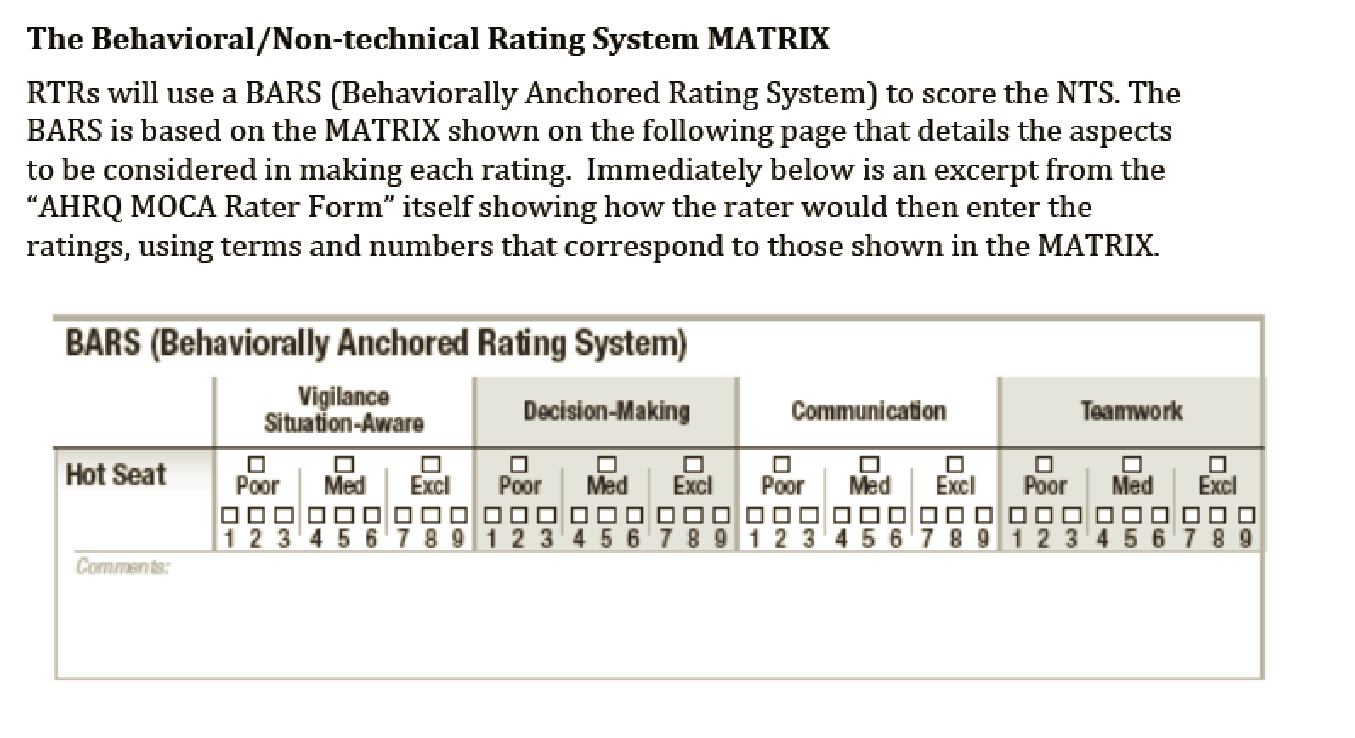

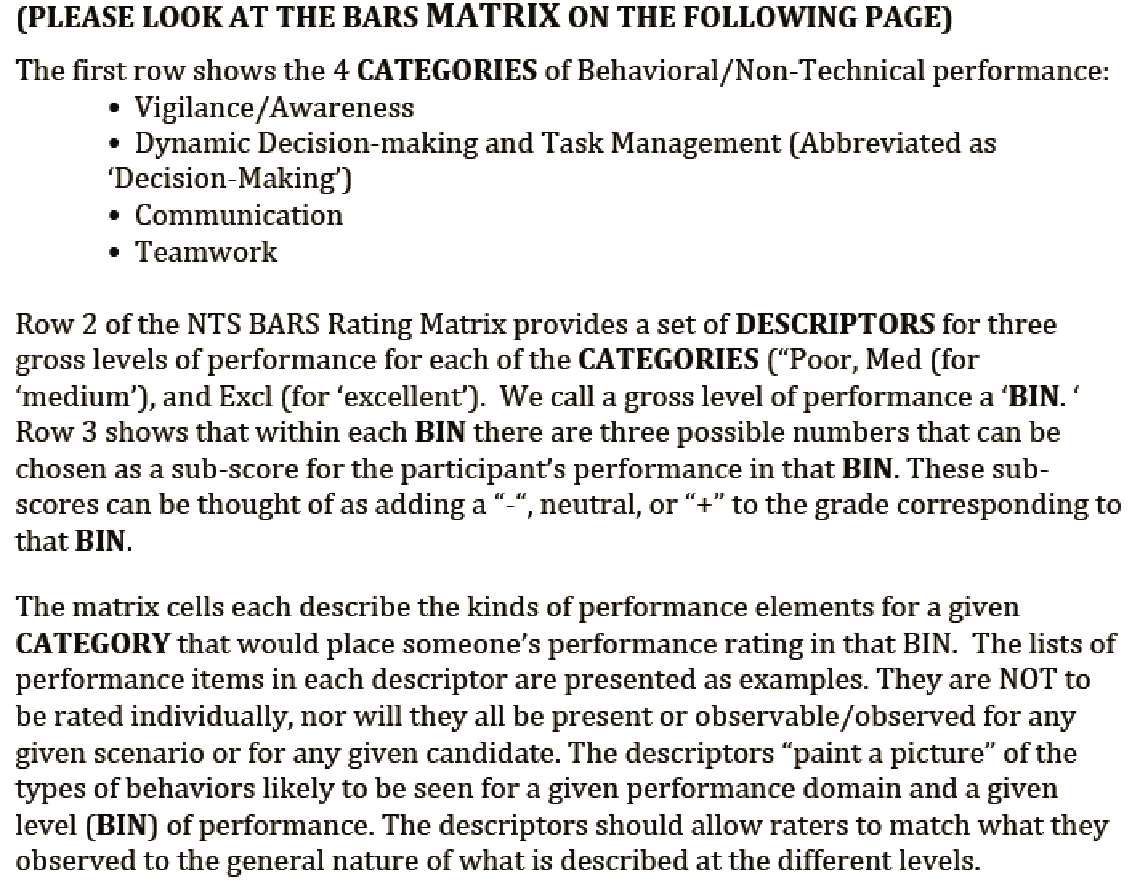


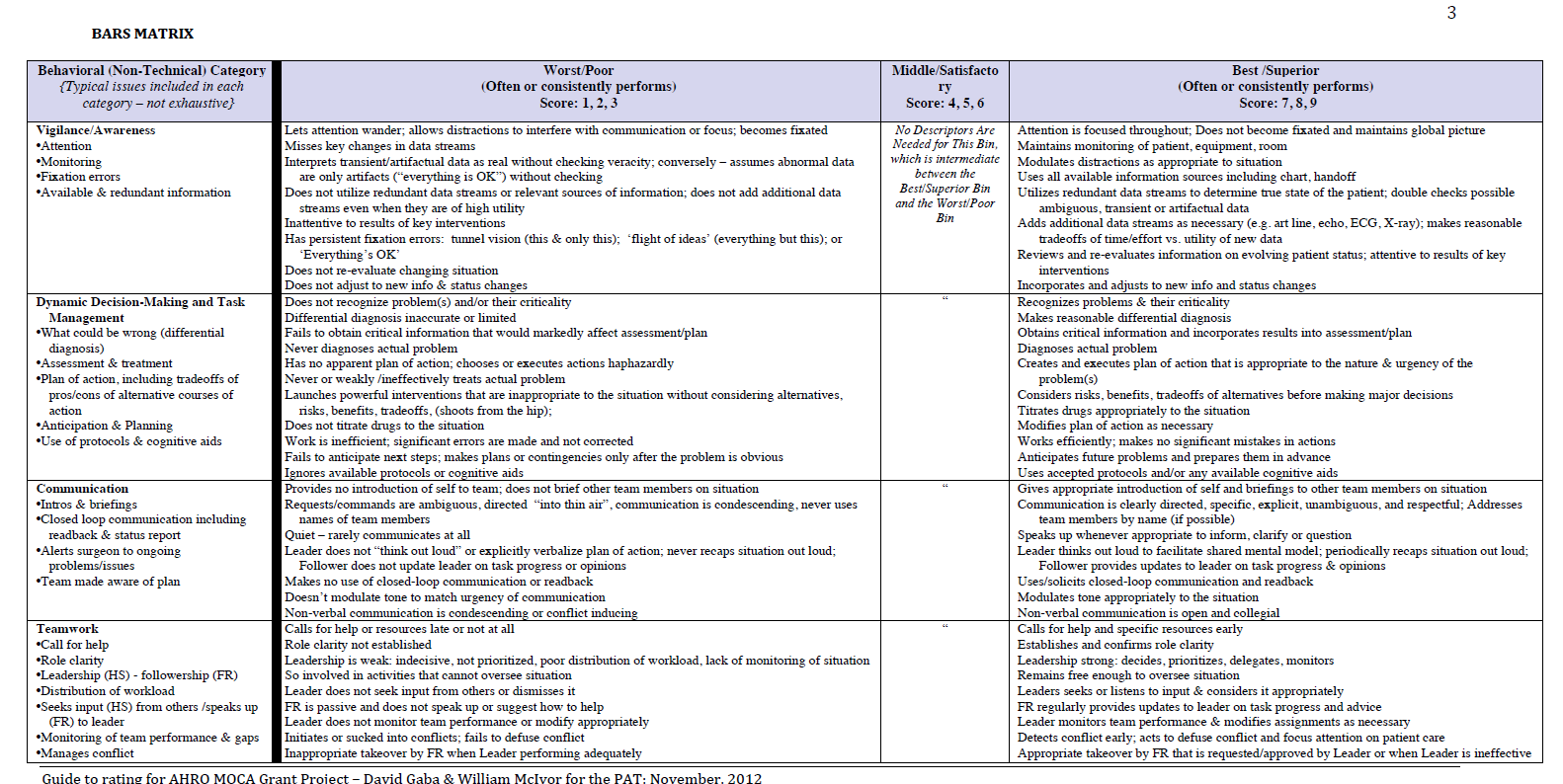


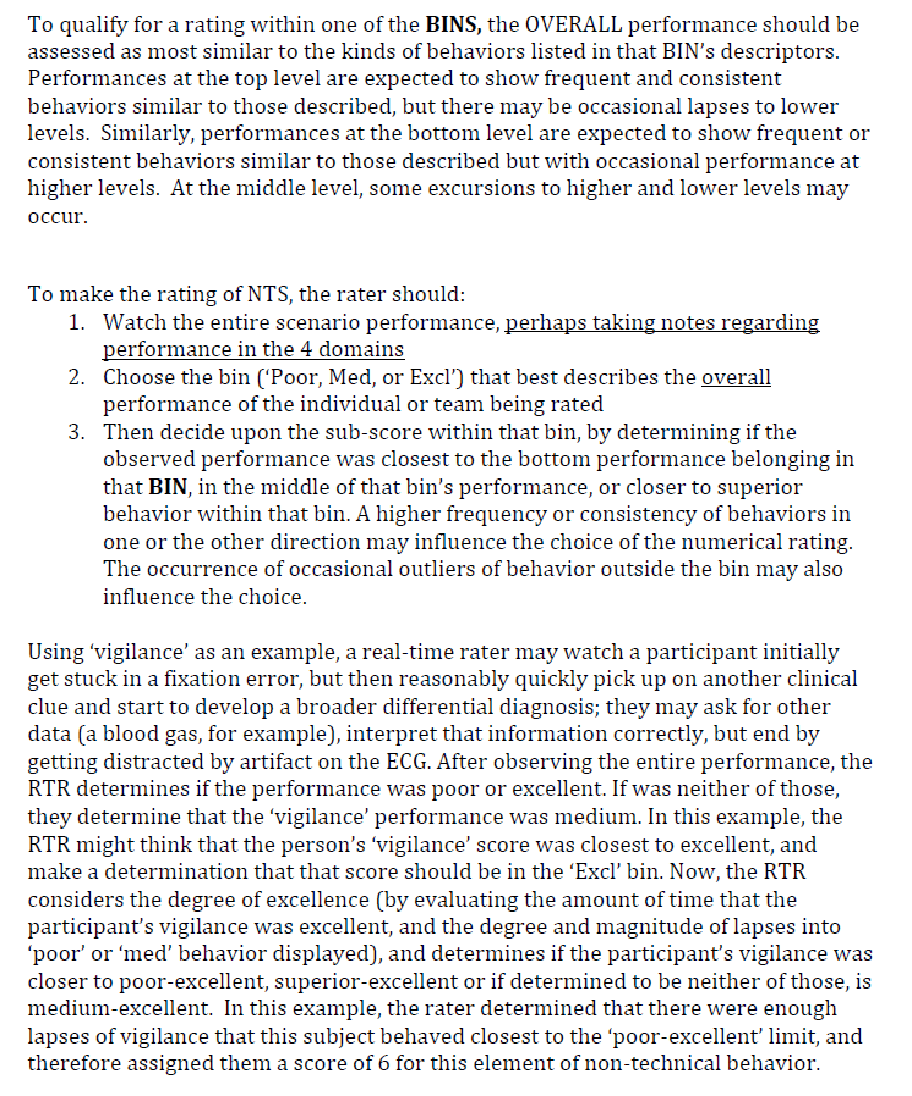


**Supplemental Material F:  System Usability Scale**


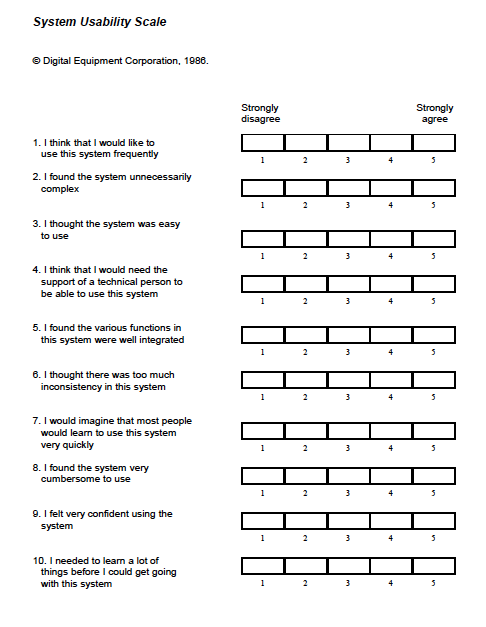


**Supplemental Material G:  Ergonomics. The ISO 9241-400 provides an assessment of user ergonomic factors.**

|  | Strongly Disagree | Disagree | Neither Agree or Disagree | Agree | Strongly Agree |
| --- | --- | --- | --- | --- | --- |
|  | 1 | 2 | 3 | 4 | 5 |
| The ML1 device is too bulky or too heavy |  |  |  |  |  |
| The mental effort (concentration) required to operate the device was very high |  |  |  |  |  |
| Arm and hands/fingers fatigue were very high |  |  |  |  |  |
| Eye fatigue was very high |  |  |  |  |  |
| Head fatigue was very high |  |  |  |  |  |
| I would be comfortable using the device for a long time |  |  |  |  |  |

ISO 9241-400 Assessment of human-ergonomic factors

**Supplemental Material H: Participant responses to the ergonomics survey (ISO 9241-400)**


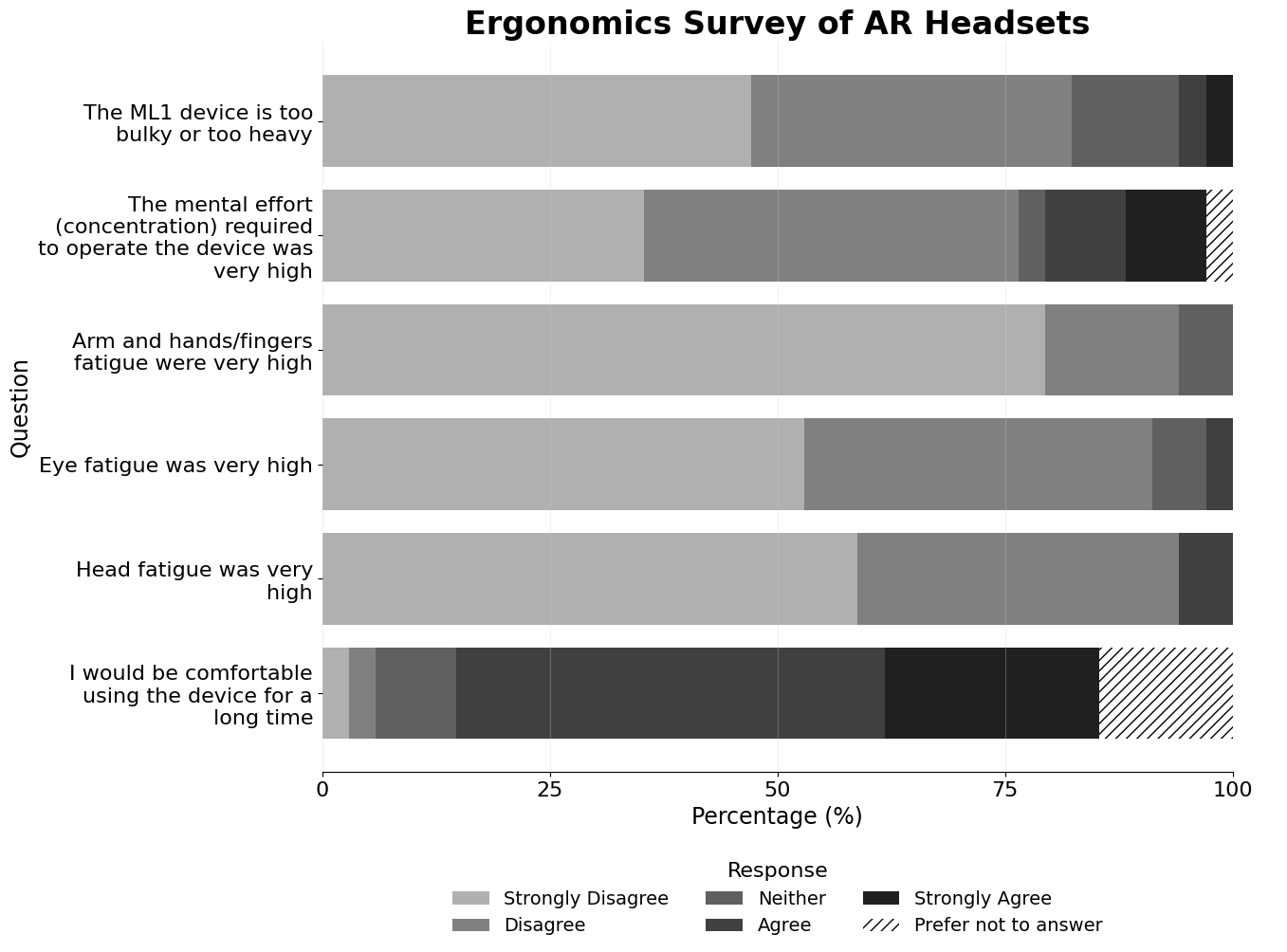

Supplement: Supplementary file 1 — Supplementary Material 1. [file 12909_2026_8611_MOESM1_ESM.docx]
